# Identifying Relapsing Fever *Borrelia*, Senegal

**DOI:** 10.3201/eid1103.040506

**Published:** 2005-03

**Authors:** Hamoud Brahim, Jean David Perrier-Gros-Claude, Danielle Postic, Guy Baranton, Ronan Jambou

**Affiliations:** * Institut Pasteur, Dakar, Senegal; † Institut Pasteur, Paris, France

**Keywords:** Spirochete, PCR, *Borrelia crocidurae*, *Borrelia recurrentis*, *Borrelia duttonii*, relapsing fever, dispatch

## Abstract

We describe a nested polymerase chain reaction for the identification of *Borrelia* species from serum of patients with unidentified fevers. This technique, based on single nucleotide polymorphisms of the 16S ribosomal RNA gene, was used to test blood samples from 7,750 patients, 33 of whom were diagnosed with spirochete infections. *Borrelia crocidurae* was the only species identified.

Tickborne relapsing fevers, caused by spirochetes of the genus *Borrelia*, vary in severity and frequency of recurrence. The prevalence of various *Borrelia* species depends on the locality. In West Africa, *Borrelia crocidurae* is the primary cause of relapsing fevers (*1*–*3*), although *B. duttonii and B. recurrentis* are also found in Senegal. Tickborne relapsing fever is difficult to diagnose and is often confused in its early stages with malaria or relapsing malaria. Diagnosis is routinely made by conventional microscopy (Giemsa-stained thick blood smear) or fluorescence microscopy after concentration of blood in capillary tubes and staining with acridine orange (quantitative buffy coat [QBC] analysis) (*3*). However, these methods require technical expertise and do not identify *Borrelia* species. Another method, culture ex vivo, is difficult to perform and rarely used in African medical laboratories. In contrast, the polymerase chain reaction (PCR) is a sensitive diagnostic tool that is widely used in developing countries in AIDS, malaria, and entomology-related disease control programs.

The objective of our study was to develop a PCR method to identify the main *Borrelia* species in uncultured serum from patients with relapsing fevers in Senegal. This study was conducted using primers for single nucleotide polymorphisms (SNPs) in the 16S ribosomal RNA (*rrs*) gene of *Borrelia* sp. Sequencing of the *rrs* gene has shown that *B. crocidurae, B. duttonii, B. recurrentis*, and *B. hispanica* belong to the same species cluster (*4*). *B. crocidurae* differs from *B. duttonii* in this gene by only 1 nt (G63A polymorphism, GenBank accession no. M88329) and from *B. recurrentis* by 2 nt (G63A and C211T, GenBank accession no. M88329) (*4*). These SNPs have also been identified in *Borrelia* species found outside sub-Saharan Africa.

We used this method to analyze blood samples collected from patients attending the medical laboratory of the Institut Pasteur in Dakar, Senegal, for suspected malaria from October 1999 to October 2003. In addition to routine laboratory analysis, PCR was performed on frozen serum. Spirochetes were first detected in fresh blood samples by microscopic examination with a QBC kit (Makromed, Johannesburg, South Africa). Five hundred microliters of serum from spirochete-positive patients was then used for extraction of DNA. Extraction was performed according to the method of Wilson (*5*), with slight modifications. Serum was diluted in distilled water (final volume 1.5 mL) and centrifuged at 15,000 rpm for 30 min at 4°C. DNA was extracted from the pellet by incubation for 2 h at 37°C in extraction buffer (1M Tris, 0.5M EDTA, proteinase K [20 mg/mL], and 10% sodium dodecyl sulfate), purified by a standard phenol/chloroform procedure (*6*), and precipitated with ethanol. Thermolysates provided by the National Leptospire Reference Center (CNRL) (Pasteur Institute, Paris, France) served as controls.

The primers used in this study are listed in the Table. *Borrelia* species were first identified by using the nested PCR reported by Ras et al. (*4*). It consisted of amplification of a 523-bp region of the *rrs* gene using primers sets fD3-T50 and Rec4-Rec9. In a second PCR, the presumptive identification of the *Borrelia* species was made using primer set Fd3-595R for the first amplification and specific primer sets (BcroF-255R for *B. crocidurae*, BdutF-255R for *B. duttonii*, and BrecF-500R for *B. recurrentis)* for the second amplification. These 3 specific primers sets were used in 3 separate reactions for the second PCR. Species identification was systematically confirmed by sequencing the *rrs* gene. The 3 species-specific primer sets include the SNPs up to their last 3′ base. Based on the alignment of sequences of *Borrelia* found in GenBank, these 3 primer sets can also hybridize with other sequences from the Eurasian *B. burgdorferi* sensu lato group and the North America relapsing fever group.

The reaction conditions were adapted to optimize species-specific amplification by using thermolysates of strains obtained from CNRL as positive controls. Serum samples negative for *Borrelia* and DNA extracted from *Escherichia coli*, *Plasmodium*, *Mycoplasma*, and *Candida* obtained from our medical laboratory were used as negative controls.

All PCR amplifications were performed in a final volume of 25 μL containing approximately 3 ng of DNA, 10 mmol/L Tris-HCl (pH 8.3), 2.5 mmol/L MgCl_2_, 200 mmol/L of each deoxynucleotide triphosphate, 1 U of *Taq* polymerase (Amersham Biosciences, Piscataway, NJ, USA), and 1 mmol/L of each primer. Amplification in all PCRs was carried out for 35 cycles with denaturation at 93°C for 1 min, annealing at 4°C below the melting temperature of the primer (Table) used for 1 min, and extension at 72°C for 2 min. For PCR species typing, annealing was performed at 70°C for 40 s and extension at 72°C for 40 s. Final PCR products were detected by electrophoresis on a 1.5% agarose gel stained with ethidium bromide. PCR analyses were performed according to Good Laboratory Practice to avoid cross-contamination between samples during extraction or amplification. Under the PCR conditions defined, the 3 West African *Borrelia* species were amplified only with their respective primers.

**Table:** Primers used for amplification of the *Borrelia* 16S ribosomal RNA gene based on GenBank accession no. M88329 for *Crocidurae*

Primer (reference)	Sequence	Tm, °C*	Nucleotide position
Fd3 (4)	AGAGTTTGATCCTGGCTTAG	58	8–27
T50 (4)	GTTACGACTTCACCCCCCT	60	1478–1499
Rec4 (4)	ATGCTAGAAACTGCATGA	50	659–675
Rec9 (4)	TCGTCTGAGTCCCCATCT	56	1191–1174
Fd4	GGCTTAGAACTAACGCTGGCAG	68	21–42
595R	CTTGCATATCCGCCTACTCA	60	621–602
500R (4)	CTGCTGGCACGTAATTAGCC	64	548–529
BcroF	CGTCTTAAGCATGCAAGTCAG	62	45–65 (U42283)
BdutF	CGTCTTAAGCATGCAAGTCAA	60	45–65 (AF107364)
BrecF	GAAAGGAAGCCTTTAAAGCTTT	60	193–214 (AF10362)
255R	CCCTACCAACTAGCTAATAAGACGC	74	255–231
*Tm, melting temperature.			

We tested potential cross-amplification of Eurasian and North American strains by using strains provided by CNRL. Typical bands were found in the thermolysates of *B. hermsii* (strainHS1), *B. parkeri* (strain M3001), and *B. turicatae* (strain 2007) when the specific primer for *B. crocidurae* was used. The thermolysates of *B. duttonii* (strain *Ly*), *B. garinii* (strain 20047), and *B. burgdorferi sensu stricto* (strain B31) were amplified with the specific primer for *B. duttonii*. However, since the geographic distribution of these pathogens does not overlap, species identification is relatively simple. For example, *B. hispanica* and *B.* *burgdorferi* sensu lato have not been previously found in sub-Saharan Africa.

Most patients attending the medical laboratory of the Institut Pasteur in Dakar, Senegal, during the study period were residents of the Dakar area. Of the 7,750 patients included in this study, 3,300 were females and 4,450 were males; their mean age was 44.5 years. A total of 605 (7.8%) patients tested positive for malaria; most infections were diagnosed as *Plasmodium falciparum*. Spirochetes were found in the blood of 33 patients by microscopic examination. Only 1 patient was infected with both spirochetes and *P. falciparum*. In contrast to malaria, detection of spirochetes does not exhibit a seasonal variation in prevalence rates. Sera were available for 25 of 33 spirochete-positive patients. Using our species-specific nested PCR, we found that serum from 25 of these patients contained *B. crocidurae.* As described earlier, the presence of this species was confirmed by gene sequencing.

This new nested PCR is an efficient method for identifying tickborne relapsing fevers in sub-Saharan Africa. The procedures were conducted over a 2-day period with standard PCR equipment and could also be useful in epidemiologic studies. This study also confirmed that *B. crocidurae* is the most prevalent *Borrelia* species in the study area.
